# Measurement of plasma norepinephrine and 3,4-dihydroxyphenylglycol: method development for a translational research study

**DOI:** 10.1186/s13104-018-3352-3

**Published:** 2018-04-19

**Authors:** Quin E. Denfeld, Beth A. Habecker, William R. Woodward

**Affiliations:** 1 Knight Cardiovascular Institute, Oregon Health & Science University, Portland, OR USA; 2 Department of Physiology & Pharmacology, Oregon Health & Science University, Portland, OR USA; 3 Department of Neurology, Oregon Health & Science University, Portland, OR USA; 4 Present Address: School of Nursing, Oregon Health & Science University, 3455 S.W. U.S. Veterans Hospital Road, Mail code: SN-ORD, Portland, OR 97239-2941 USA

**Keywords:** Sympathetic nervous system, Norepinephrine, 3,4-dihydroxyphenylglycol, High performance liquid chromatography, Electrochemical detection, Human plasma

## Abstract

**Objective:**

Norepinephrine (NE), a sympathetic neurotransmitter, is often measured in plasma as an index of sympathetic activity. To better understand NE dynamics, it is important to measure its principal metabolite, 3,4-dihydroxyphenylglycol (DHPG), concurrently. Our aim was to present a method, developed in the course of a translational research study, to measure NE and DHPG in human plasma using high performance liquid chromatography with electrochemical detection (HPLC-ED).

**Results:**

After pre-purifying plasma samples by alumina extraction, we used HPLC-ED to separate and quantify NE and DHPG. In order to remove uric acid, which co-eluted with DHPG, a sodium bicarbonate wash was added to the alumina extraction procedure, and we oxidized the column eluates followed by reduction because catechols are reversibly oxidized whereas uric acid is irreversibly oxidized. Average recoveries of plasma NE and DHPG were 35.3 ± 1.0% and 16.3 ± 1.1%, respectively, and there was no detectable uric acid. Our estimated detection limits for NE and DHPG were approximately 85 pg/mL (0.5 pmol/mL) and 165 pg/mL (0.9 pmol/mL), respectively. The measurement of NE and DHPG in human plasma has wide applicability; thus, we describe a method to quantify plasma NE and DHPG in a laboratory setting as a useful tool for translational and clinical research.

**Electronic supplementary material:**

The online version of this article (10.1186/s13104-018-3352-3) contains supplementary material, which is available to authorized users.

## Introduction

As the principal sympathetic neurotransmitter, norepinephrine (NE), plays a critical role in regulating physiological processes [1, 2] and is commonly used as an index of sympathetic activity in healthy and diseased states [3–5]. In some conditions (e.g. heart failure) there is a noted increase in plasma NE [6] as a result of increases in sympathetic activity and subsequent NE “spillover” from synapses into the plasma [7, 8] as well as reduced reuptake of NE [9]. Elevated plasma NE portends worse outcomes such as worsening left ventricular function [10] and mortality [7, 11]. There is wide variation, however, in approaches to measure plasma NE [12–16]. Two common approaches involve either radioenzymatic [13] or enzyme immunoassay methods [14]. While these methods are well validated, they either require significant experimental considerations (e.g. use and disposal of radioisotopes) or they are limited to the measurement of NE alone. To gain a better sense of NE dynamics, measuring its principal metabolite, 3,4-dihydroxyphenylglycol (DHPG), provides insight into NE dynamics, offering an index of NE turnover [17–19]. As such, the measurement of both plasma NE and DHPG provides unique and complementary information about sympathetic activity in conjunction with other metrics. Our aim was to present a method, developed in the course of a translational research study, to measure NE and DHPG in human plasma using high performance liquid chromatography with electrochemical detection (HPLC-ED). This method has the benefit of a simple sample preparation process and concurrent measurement of both NE and DHPG, as well as being high-throughput.

## Main text

### Materials and methods

#### Plasma sample and standard preparation

We processed samples that were previously collected from heart failure patients as part of a National Institutes of Health-funded study [20]. Whole blood was centrifuged at 1000×*g* for 10 min at 5 °C to extract the plasma; the plasma was then de-identified as part of a biorepository and frozen at − 80 °C. When ready to process, frozen plasma samples were thawed and then centrifuged at 8000×*g* for 3 min at 4 °C to remove insoluble material. Plasma samples (volumes ranging from 200 to 500 μL, depending on availability) or standards (500 μL; containing 0.1 μM NE and DHPG in distilled, deionized water (ddH_2_O)) were mixed with 250 μL of 0.2 M perchloric acid (PCA) containing 0.2 μM dihydroxybenzylamine (DHBA; internal standard described below) and ddH_2_O, if necessary, to make the final volume 1 mL. An aliquot of the supernatant (700 μL) was combined with 300 μL of 3.0 M Tris, pH 8.5 containing 0.1 mM EDTA and 15 mg of alumina (Activity Grade Super I; ICN Biomedicals). The samples were tumbled for 15 min. The supernatant was aspirated from the alumina, and the alumina was washed once with 1 mL of 0.2 M sodium bicarbonate and twice with 1 mL of ddH_2_O (with a vortex-mix and 10 s centrifugation between washes). Following the final water wash, 0.1 M PCA (150 μL) was added to the alumina to desorb the catechols. A 50 μL aliquot of the sample was injected onto the HPLC column for analysis.

#### High performance liquid chromatography

The catechols were separated by reversed-phase chromatography on C18 column (Agilent Microsorb, 150 × 4.6 mm, 5 μm) using a filtered and degassed mobile phase consisting of 75 mM sodium phosphate (pH 3.0), 1.7 mM sodium octane sulfonate, 1.5% acetonitrile with a flow rate of 1.0 mL/min. The mobile phase was maintained with a Shimadzu L10AD pump, and a Shimadzu SIL-20AC HT autosampler was used to inject 50 μL aliquots of either sample or standard.

#### Electrochemical detection

**Fig. 1:**
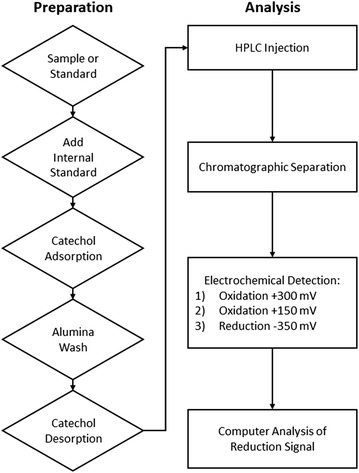
Flow diagram for the oxidation–reduction protocol measuring NE and DHPG in plasma. First, to each of the samples or standards, an internal standard (i.e. DHBA) is added. Then, the catechols (NE, DHPG, and DHBA) are adsorbed onto alumina. The alumina is washed first with sodium bicarbonate and then twice with water to remove any substances not bound to the alumina. The catechols are desorbed from the alumina using 0.1 M PCA. An aliquot of the supernatant is injected onto the HPLC, and the catechols are separated by liquid chromatography on the HPLC. The catechols are first oxidized at + 300 and + 150 mV and then reduced at − 350 mV. The output of the reduction signal is analyzed by the computer software. *DHBA* dihydroxybenzylamine, *DHPG* 3,4-dihydroxyphenylglycol, *HPLC* high performance liquid chromatography, *NE* norepinephrine, *PCA* perchloric acid

An electrochemical detector (ESA Coulochem III; Bedford, MA) was used to detect and quantify the catechols. When using the oxidation protocol (detector set at +180 mV) in test plasma runs, there was a large peak that co-eluted with DHPG that we identified as uric acid. Even though uric acid is poorly adsorbed onto alumina, the high amounts of uric acid that are present in the plasma of heart failure patients [21, 22] result in peaks that are much larger than those for DHPG. We took advantage of the reversible oxidation of catechols but irreversible oxidation of uric acid under our conditions to analyze catechols in the plasma samples. Thus, we used an oxidation–reduction protocol in which a conditioning electrode was set at + 300 mV to oxidize all analytes in the eluate, the first analytical electrode was set at + 150 mV to insure complete oxidation of all analytes, and the second analytical electrode set at − 350 mV to reduce the analytes [23]. The gain on the reducing detector was set at 50 nA full-scale. LabSolutions (Shimadzu) software was used to collect and analyze the data (see Fig. 1 for flow diagram).

#### Calculation of results

The internal standard method is commonly acknowledged to be the gold standard in quantifying analytes in complex mixtures such as plasma [24]. Since the internal standard, DHBA, behaves similarly to the plasma catechols in the alumina extraction and electrochemical detection process (but is not found in biological samples), it is possible to quantify plasma catechols in samples by referring them to standards, which both contain a standard amount of DHBA. Using the ratio of the plasma catechol peak area (i.e. NE or DHPG) to the DHBA peak area, divided by a similar ratio for the catechol standard, this ratio of ratios is then adjusted for the fraction of the sample used in the assay [correction factor (CF)] by the following equation to obtain the catechol concentration per mL of plasma.

$$\frac{{\left\{ {\frac{{Catechol_{sample} }}{{DHBA_{sample} }}} \right\}}}{{\left\{ {\frac{{Catechol_{standard} }}{{DHBA_{standard} }}} \right\}}}\varvec{ } \times CF\varvec{ } = Catechol\;per\;mL\;plasma$$

#### Statistical analysis

Data were analyzed using means and standard deviations. We used linear regression analysis to establish the linearity and variability of the method. All analyses were performed using GraphPad Prism version 7.02.

### Results

To quantify linearity of the method, we analyzed a range of concentrations of NE (0–0.12 μM), DHPG (0–0.18 μM), and DHBA (0–0.12 μM) that reflected the expected range of these compounds in plasma using alumina extraction and an oxidation–reduction protocol. The peak areas of NE and DHPG relative to DHBA were compared (i.e. NE:DHBA and DHPG:DHBA, respectively). The method was linear for NE (r^2^ = 0.997) and DHPG (r^2^ = 0.983) over this range of concentrations (Additional file 1: Figure S1).

To quantify recovery amounts, we processed known concentrations of a standard mix (containing 0.5 μM DHPG, 0.5 μM NE, and 0.5 μM DHBA) and uric acid alone (50 μM). Using both oxidation and oxidation–reduction protocols, we processed the standard mix (*n *= 6) and uric acid (*n *= 6) with and without alumina extraction. Average recoveries are reported in Table 1. Despite removing > 99% of uric acid with alumina extraction and an oxidation protocol, the remaining uric acid still yielded a peak (recovery ~ 0.075 μM) that overlaid the DHPG peak (recovery ~ 0.155 μM). Alumina extraction coupled with the oxidation–reduction protocol eliminated the uric acid while maintaining adequate recoveries of NE and DHPG.

**Table 1:** Recoveries following alumina extraction (*n *= 6)

	M ± SD	
	Oxidation^a^	Oxidation followed by reduction^a^
DHPG	31.0 ± 0.8%	16.3 ± 1.1%
Uric acid	0.15 ± 0.01%	ND
Norepinephrine	49.3 ± 1.2%	35.3 ± 1.0%
DHBA	45.4 ± 1.1%	32.1 ± 0.8%

Comparison of recoveries of processed samples using the oxidation and oxidation–reduction protocols. Standard mixes (*n *= 6; each containing 0.5 μM DHPG, 0.5 μM NE, and 0.5 μM DHBA) and uric acid (*n *= 6; each containing 50 μM) were processed and subjected to either the oxidation protocol or the oxidation–reduction protocol. We calculated recoveries of each compound based on the starting amount without alumina extraction. Even though uric acid was poorly adsorbed onto alumina, the concentration of uric acid in plasma is high compared with the catechols and even a small percentage retained obscures the DHPG peak. *DHBA* dihydroxybenzylamine, *DHPG*, 3,4-dihydroxyphenylglycol, *M* mean, *ND* non-detectable, *SD* standard deviation

^a^Recoveries are expressed as a percentage of the starting amount

**Fig. 2:**
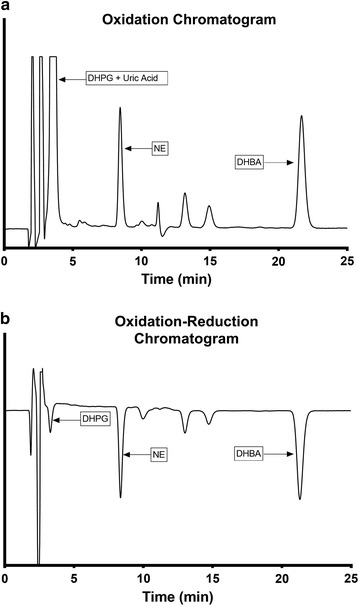
Representative chromatograms of plasma samples using the oxidation protocol (**a**) or oxidation–reduction protocol (**b**). Each plasma sample was pre-purified with alumina extraction and processed with high performance liquid chromatography. Plasma samples were then either oxidized using detector settings of +180 mV (**a**) or oxidized and then reduced using detector settings of + 300, + 150, and − 350 mV (**b**). *DHBA* dihydroxybenzylamine, *DHPG* 3,4-dihydroxyphenylglycol, *NE* norepinephrine

Representative chromatograms from plasma samples are shown in Fig. 2. In Fig. 2a, using oxidation alone, there was a large uric acid peak that co-eluted with DHPG. By including a sodium bicarbonate wash of the alumina and an electrochemical protocol involving oxidation followed by reduction, the co-eluting peak of uric acid was eliminated (Fig. 2b), unmasking the underlying DHPG peak. The peaks are negative due to the absorption of electrons by the compounds as a result of reduction. The average intra-assay coefficient of variation was 5.3% (*n *= 35–40), and the inter-assay coefficient of variation was 4.6% (*n *= 4). Based on a plasma sample of 500 μL, our estimated detection limit was approximately 85 pg/mL (0.5 pmol/mL) for NE and 165 pg/mL (0.9 pmol/mL) for DHPG, using a signal/noise ratio of ≥ 3.

### Discussion

Building on previous methods [23, 25], we describe a method to measure plasma NE and its principal metabolite, DHPG, that was developed as part of a translational research study. Our main findings are: (1) alumina extraction coupled with an electrochemical detection protocol involving oxidation followed by reduction eliminated a uric acid contaminant that co-eluted with DHPG, and (2) we were able to quantify NE and DHPG in plasma samples with reasonable detection limits.

An advantage of this method is the measurement of the direct principal metabolite, DHPG, which yields important information above and beyond the measurement of NE. DHPG is closer to NE in the metabolic scheme than the end metabolite, vanillylmandelic acid [18] and reflects the neuronal metabolism of NE compared with the non-neuronal metabolite, normetanephrine [1]. Importantly, DHPG can be measured in the same assay as NE. Our findings demonstrate that a simple set-up with HPLC-ED, including an alumina extraction coupled with a sodium bicarbonate wash and an oxidation–reduction protocol, may rapidly increase the feasibility for clinical laboratories to detect DHPG in addition to NE.

Further consideration relates to the quantity of human plasma required for NE and DHPG detection. Human plasma is often of limited quantity and must be carefully allocated for various assays and experiments. Complicating this circumstance, catecholamines exist in small quantities in biological fluids, which demands that bioanalytical methods must be specific and sensitive enough to detect these small quantities. In our application, we were able to detect and easily quantify amounts of NE and DHPG in plasma samples as low as 200 μL.

Our intent with this paper was to demonstrate how to measure plasma NE and DHPG with applicability to both translational and clinical research studies. While there are noted benefits and drawbacks to the various methods to measure plasma NE [14, 15, 24, 26], researchers frequently do not have access to extensive set-ups in laboratories nor the expertise to perform complicated assays. Moreover, plasma may be the only biological fluid available, as opposed to multiple-hour urine collection [27], for example. This described method has the advantage of being simple and can be set-up in any laboratory that has HPLC-ED. We outline the necessary parameters, including the sample preparation process and the chromatographic and detector settings, which permit concentration, separation, and quantification of the compounds of interest. Finally, this method was high-throughput; we were able to process about 40 samples in 2 days. In conclusion, there are multiple applications within translational and clinical research for HPLC-ED measurement of NE and its principal metabolite, DHPG, yielding clinically significant information on sympathetic activity and contributing to translational knowledge regarding key physiological processes in both health and disease.

## Limitations

There are few noted limitations to this method, including the acknowledgment of well-documented analytical challenges [1, 24, 28]. First, even though we were able to estimate the neuronal reuptake of NE with DHPG levels, this method does not permit the estimation of the non-neuronal clearance of plasma NE nor the kinetics of NE. Second, we used samples collected from the forearm, which does not necessarily reflect sympathetic activity in the rest of the body because sympathetic outflow varies among tissues and organs [1]. Finally, the recovery of DHPG in the oxidation–reduction protocol was lower than NE possibly due to the sodium bicarbonate wash or less efficient reduction of DHPG. Future work to improve this method should involve developing techniques to increase the recovery of DHPG, particularly when the absolute concentration is necessary. Additionally, these methods should be compared with other known methods using the same plasma samples to quantify differences in reported concentrations.

## Additional file

**Additional file 1: Figure S1.** Linearity of the response for NE (A) and DHPG (B) based on a range of physiological concentrations compared with the ratio of NE or DHPG to the internal standard, DHBA. *DHBA* dihydroxybenzylamine, *DHPG* 3,4-dihydroxyphenylglycol, *NE* norepinephrine.

## Abbreviations

CF: correction factor
ddH_2_O: distilled, deionized water
DHBA: dihydroxybenzylamine
DHPG: 3,4-dihydroxyphenylglycol
HPLC-ED: high performance liquid chromatography with electrochemical detection
NE: norepinephrine
PCA: perchloric acid
