# Supplementary figures and images for: Measurement of plasma norepinephrine and 3,4-dihydroxyphenylglycol: method development for a translational research study
Source: BMC Res Notes. 2018 Apr 19;11:248. doi: 10.1186/s13104-018-3352-3 (PMC5909231; doi:10.1186/s13104-018-3352-3)

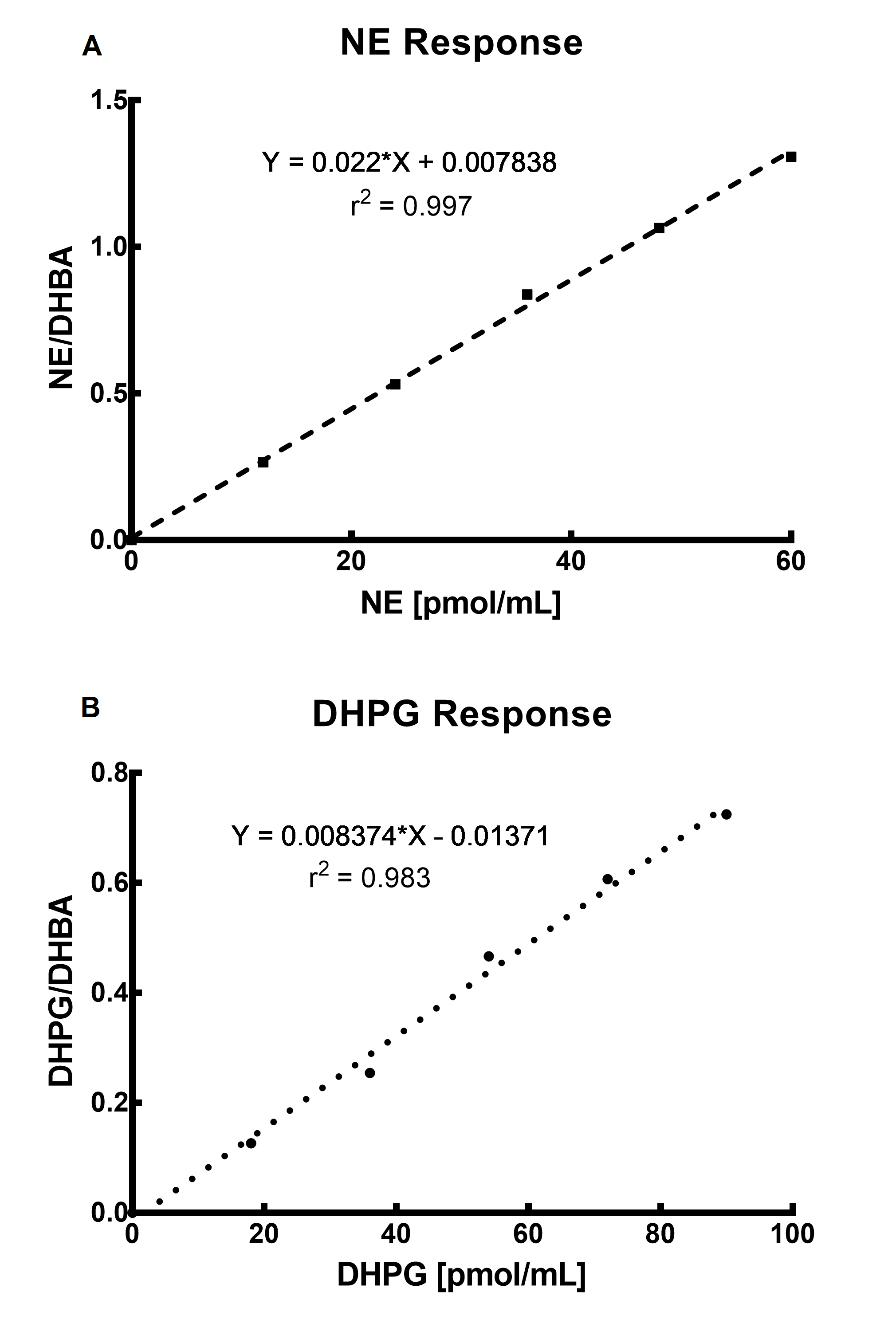

Supplement: Supplementary file 1 — Additional file 1: Figure S1. Linearity of the response for NE (A) and DHPG (B) based on a range of physiological concentrations compared with the ratio of NE or DHPG to the internal standard, DHBA. DHBA dihydroxybenzylamine, DHPG 3,4-dihydroxyphenylglycol, NE norepinephrine. [file 13104_2018_3352_MOESM1_ESM.tif]
